# An exploratory mixed-methods study using the ELICIT framework to identify stakeholder-informed implementation strategies for a population health platform

**DOI:** 10.1186/s12913-026-14723-8

**Published:** 2026-05-25

**Authors:** Anne C. Madeo, Jarrod Marable, Polina Kukhareva, Wendy K. Kohlmann, Richard L. Bradshaw, Whitney Maxwell, Che Martin, Lauren Davis-Rivera, Muhammad Danyal Ahsan, Emerson P. Borsato, Elena B. Elkin, Kimberly A. Kaphingst, Melissa K. Frey, Ravi N. Sharaf, Kensaku Kawamoto, Guilherme Del Fiol, Chelsey Schlechter, Caitlin G. Allen

**Affiliations:** 1 Department of Population Health Sciences, Spencer Fox Eccles School of Medicine, University of Utah, Salt Lake City, UT USA; 2 Huntsman Cancer Institute, University of Utah, Salt Lake City, UT USA; 3 Department of Implementation Science, Wake Forest University School of Medicine, Winston Salem, NC USA; 4 Department of Biomedical Informatics, Spencer Fox Eccles School of Medicine, University of Utah, Salt Lake City, UT USA; 5 Department of Informatics, New York Presbyterian Hospital, New York, NY USA; 6 Genetics and Personalized Cancer Prevention Program, Weill Cornell Medicine, New York, NY USA; 7 Department of Health Policy and Management, Columbia University Mailman School of Public Health, New York, NY USA; 8 Department of Population Health Sciences, Weill Cornell Medicine, New York, NY USA

**Keywords:** Humans, Information technology, Focus groups, Population health management, Testing, genetic predisposition, Genetic predisposition to disease, Implementation science, Electronic health records

## Abstract

**Background:**

GARDE is an open-source software platform that supports population health management interventions including hereditary cancer cohort identification, patient engagement, education, and access to genetic testing services. To facilitate GARDE implementation, we applied the Evaluation in Life Cycle of Information Technology framework to 1) identify barriers and facilitators to the implementation of GARDE at five healthcare systems in differing implementation phases, 2) develop implementation strategies to address barriers, and 3) prioritize implementation strategies for implementation toolkit development based on partner-rated feasibility and importance.

**Methods:**

We facilitated focus group discussions and individual in-depth interviews (*n* = 17) at five healthcare systems that have implemented or are implementing GARDE to identify contextual determinants of implementation and strategies to address implementation barriers. We relied on the Evaluation in Life Cycle of Information Technology framework to develop the interview guide and analyze data using rapid qualitative techniques. Our team identified implementation strategies in response to reported barriers. GARDE implementers were surveyed to assess the feasibility and importance of implementation strategies.

**Results:**

Participants at all healthcare systems identified multiple GARDE implementation barriers and facilitators, largely focusing on information technology needs to effectively implement an electronic health record-integrated innovation. Eight implementation strategies were identified as both important and feasible to address identified barriers.

**Conclusions:**

Population health management innovations are a potential solution to healthcare system challenges through cohort identification, patient engagement, and access to relevant healthcare services, but their implementation must meet multiple requirements. This study of an EHR-integrated population health management platform implementation used an Evaluation in Life Cycle of Information Technology -grounded approach to identify implementation barriers and facilitators and implementation strategies to address barriers. An implementation toolkit is being refined to support the implementation and dissemination of population health management platforms.

**Supplementary Information:**

The online version contains supplementary material available at 10.1186/s12913-026-14723-8.

## Background

Evidence-based guidelines recommend that clinicians collect patients’ cancer family history to offer risk-stratified prevention best-suited to the individual [1–9]. A solution to primary care clinician concerns about this task [10–13] is to automate risk stratification and outreach to patients at the population level, leveraging electronic health record (EHR) data, and pair patient identification with automated patient education and outreach. GARDE is an EHR-integrated population health management platform that was initially developed to improve health system-level identification of individuals at an increased risk of hereditary cancer and facilitate patient engagement, education, and access to genetic testing. GARDE screens cancer family history in the EHR to identify individuals without cancer who meet criteria for genetic evaluation for hereditary breast, ovarian, prostate, pancreatic, and/or colorectal cancers [14–16] and offers a chatbot authoring platform that facilitates the creation of patient-directed chatbots for outreach, education, and testing access.

To be successful, population health management innovations must be integrated into an existing EHR system, meet the clinical needs of an organization, be acceptable to users, align with existing clinical workflows and information systems, and be portable across health systems [17–19]. Studies of EHR-integrated patient-based screens have described challenges to their implementation, including variations in how providers use the EHR and communicate with patients, limited staff capacity to address concerns during patient visits, staff knowledge and self-efficacy regarding the management of conditions, financial and clinic personnel constraints [20, 21]. These findings underscore the importance of evaluating the complexities of implementing GARDE to facilitate the implementation of EHR-integrated innovations within healthcare systems. To facilitate our understanding of these complexities, the research was informed by the Evaluation in Lifecycle of Information Technology (ELICIT) framework [22]. We selected the ELICIT framework because it was developed to encompass the full life cycle of electronic health record (EHR)-integrated innovations and the varied levels that are affected by and affect EHR integrated innovations (society, user and software).

Previous research has investigated aspects of EHR-integrated innovations, including their development [23–25] and implementation [23, 25, 26]. However, these studies have rarely described the identification of implementation strategies [27, 28] to facilitate their integration into other health systems. When research has described the development of implementation strategies their development is usually atheoretical [25, 27] or relies on implementation science frameworks that were not specifically developed to address the unique challenges of implementing an EHR-integrated innovation [29, 30]. This research is one of the first to develop implementation strategies for an EHR-integrated innovation based on the ELICIT framework.

The objectives of this study were to 1) apply the ELICIT framework to identify GARDE implementation barriers and facilitators at five healthcare systems, 2) develop implementation strategies to address identified barriers using a systematic, ELICIT-informed approach [31], and 3) prioritize implementation strategies based on partner-assessed feasibility and importance. The results of this study will be used to guide the design of a GARDE implementation toolkit and may help implementers of other EHR-integrated population health management innovations.

## Methods

**Fig. 1:**
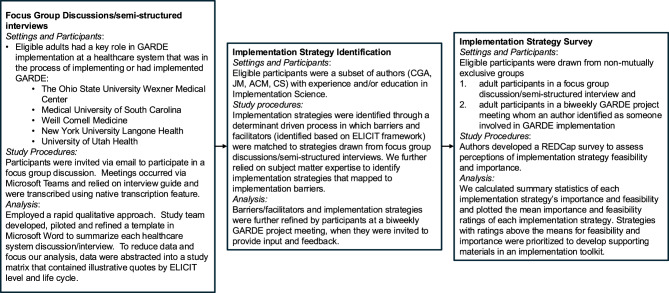
Graphical overview of methods

Study methods included focus groups, individual interviews, and surveys with GARDE partners at five healthcare systems that are implementing or have implemented GARDE. A graphical representation of the Methods can be seen in Fig. 1. In developing this study and writing this manuscript, we used the Planning for and Assessing Rigor in Rapid Qualitative Analysis framework [32]. Further, in writing this manuscript we relied on the Consolidated Criteria for Reporting Qualitative Studies [33].

### Ethics approval

This study was reviewed and determined by the University of Utah Institutional Review Board to be non-human subjects research (IRB_00176563). Thus, the need for consent to participate was waived. The research team obtained permission, but not informed consent, from all focus group attendees/interviewees to participate in and record the discussions. Stakeholders were invited to complete the anonymous Implementation Strategy Survey via an emailed cover letter that did not include a formal consent.

### Theoretical framework

**Fig. 2:**
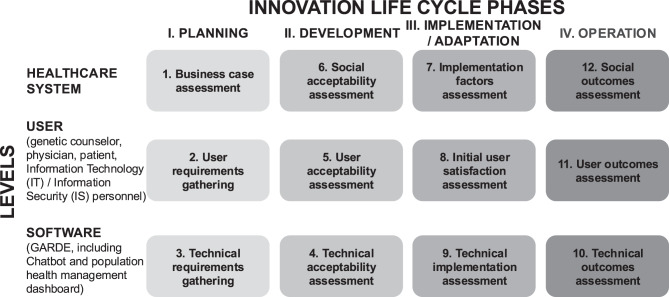
Evaluation in life cycle of IT (ELICIT) framework as applied in this study (adapted from Kukhareva et al. (2022)

Given the complexity associated with EHR-integrated innovations, we used the ELICIT framework [22] (Fig. 2) when developing the interview guide, template summaries, analyzing our data, and developing tailored implementation strategies (the “methods or techniques used to enhance the adoption, implementation, and sustainability of a clinical program or practice” [31]). ELICIT was selected because it was developed as an EHR-integrated innovation evaluation framework. It considers each phase of the IT life cycle and the multiple levels that are affected by and affect EHR-integrated innovations. For this use case, we defined life cycles and levels within each cycle as shown in Table S1 (Supplementary Material).

### Settings and participants

*Focus Group Discussions/semi-structured interviews*: This study relied on a purposive sampling strategy in which eligible adults were identified by an author as someone with a key role in GARDE implementation at a healthcare system that was in the process of implementing or had implemented GARDE (The Ohio State University Wexner Medical Center (OSUWMC), Medical University of South Carolina (MUSC), Weill Cornell Medicine (WCM), New York University Langone Health (NYULH), University of Utah Health (UTHealth)). OSUWMC, MUSC, WCM, NYULH, and UTHealth first ran GARDE algorithms assessing hereditary cancer risk at their sites in October 2024, March 2024, May 2024, January 2021, and September 2018, respectively. OSUWMC, NYULH and UTHealth implemented GARDE under a research protocol; WCM and MUSC did not.

*Implementation Strategy Identification and Development*: Four authors (CGA, ACM, JM, CRS) with experience and/or education in implementation science identified implementation strategies in analyzed transcripts and developed additional strategies.

*Implementation Strategy Survey*: Eligible participants were drawn from non-mutually exclusive groups: 1) adult participants in a focus group or an interview and 2) adult participants in a biweekly GARDE project meeting whom an author identified as someone involved in GARDE implementation [34]. Attendees at the GARDE project meeting included multi-level GARDE implementation partners (e.g., site principal investigator, genetic counselor, study staff) from MUSC, UTHealth, and WCM.

### Study Procedures

#### Data collection

*Focus Group Discussions/semi-structured interviews*: Participants were recruited via email invitation from an author. All focus group discussions/semi-structured interviews occurred via Microsoft Teams (Teams) and relied on the same interview guide (see Supplementary Materials). Because meetings were held virtually, participants were not required to be in a particular setting (e.g. home, office) when they participated in the focus group/interviews and the research team did not ask participants to explain where they were or if anybody was with them. The interview guide was developed by the interdisciplinary study team based on a content analysis orientation, important constructs in ELICIT and the implementation of an evidence-based practice [22, 35] and was not pilot tested.

Focus groups/interviews were facilitated by an author (CGS, ACM, CRS) with experience and/or graduate coursework in in-depth interviewing and focus group facilitation. In some cases, the facilitator/interviewer had an existing relationship with participant(s) due to mutual participation in a biweekly GARDE project meeting. When possible, a notetaker from the research team attended and summarized discussion content. All implementation partners from a single healthcare system participated in a single focus group, when possible. The number of participants/focus group ranged from 3 to 4. Data from two healthcare systems were obtained through semi-structured interviews with individual participants. No repeat focus group discussions/interviews were conducted.

Focus group discussions and interviews were recorded and transcribed using Teams’ native transcription feature. An author verified each transcript’s accuracy; identified errors were aligned with the original audio. All transcripts were deidentified.

*Reflexivity Statement*: As an interviewer and focus group facilitator, ACM is a cisgender woman whose background as a genetic counselor, current PhD training in population health science and knowledge of GARDE informed her communication style and interpretation of participant perspectives. Similarly, CGA and CRS are cisgender women with backgrounds as PhD implementation and behavioral scientists and knowledge of GARDE. This background informed their communication style and interpretation of participant perspectives; JM is a cisgender man whose background as a Program Manager in the Wake Forest University School of Medicine Precision Health Research Laboratory and knowledge of GARDE informed his interpretation of participant perspectives. CGA, CRS, and ACM are regular participants in bi-weekly GARDE project meetings, which increased their familiarity with the study context and some of the participants. This may have shaped the assumptions brought to data collection and analysis, as well as the information shared by the facilitator/interviewer. We remained attentive to these influences through ongoing discussions with the research team and attempted to address some of them by ensuring that focus group discussion facilitators/interviewers were not employed by the same healthcare system as the participant(s).

*Implementation Strategy Identification:* CGA, ACM, JM and CRS identified implementation strategies through a determinant driven process in which barriers and facilitators (identified based on ELICIT framework, see Fig. 2) were matched to discrete strategies drawn from the study matrix. We further relied on subject matter expertise to identify strategies that mapped to implementation barriers identified by focus group/interview participants.

*Implementation Strategy Survey*: We developed a survey (see Supplementary Materials) to assess the feasibility and importance of each implementation strategy. The survey was administered via REDCap (Research Electronic Data Capture) eligibility survey, hosted at the University of Utah [36–38]. It provided definitions of importance and feasibility and asked respondents to rate each strategy’s importance (“the impact of the implementation strategy and how critical it is to successful implementation of GARDE at your organization”) and feasibility (“the extent to which an implementation strategy is practical and can be successfully used to support the implementation of GARDE at your organization”). Participants were encouraged to use the full range of values across their assessments. Importance and feasibility were criteria selected to evaluate each implementation strategy because together they are expected to affect perceptions of implementation strategy acceptability and ultimately, the likelihood of strategy use by implementation partners [39].

### Analysis

*Focus Groups/interviews*: We analyzed focus group and interview data using a rapid qualitative approach [40] because it encourages the use of embedded researchers with working knowledge of contexts and topics [40], and individuals with high “information power” [41] were available as participants. The study team created and piloted a transcript summary template in Microsoft Word. After piloting the template, the study team met via videoconferencing software to ensure consistency in capturing domains. The study team relied on informal discussion to achieve consensus (unanimity in perspective). The template was modified, and the revised template summary was used by three individuals (CGA, ACM, JM) to summarize each healthcare system’s discussion, including detailed quotes [42]. The study team developed a study matrix in Microsoft Excel to reduce data and focus analysis. The study matrix was populated by a single individual (CRS) with illustrative quotes from template documents [43]. The study team met to review the completed study matrix and used it to consider and summarize differences in implementation at the ELICIT level. As we analyzed the data we did not consider data saturation, or “the point at which no new themes or codes ‘emerge’ from the data” [44], because we were not engaging in deductive coding and analysis of our data and, thus, did not expect the codebook to change with additional participants. Because we relied exclusively on the predetermined framework for analyzing transcripts, we did not code transcript data that fell outside of that framework. Transcripts, templates and the study matrix were not shared with participants, although to enhance the validity of our findings and corroborate the study team’s understanding of focus group participants’/interviewees’ quotes, feedback on our analysis was solicited from participants at a biweekly GARDE project meeting [45], held via Teams.

*Implementation Strategy Survey*: We calculated summary statistics of each implementation strategy’s importance and feasibility and plotted the mean importance and feasibility ratings of each implementation strategy [46]. The scatterplot was divided into four quadrants using the importance and feasibility means as the midpoint of each axis. Strategies found in Quadrant 1 (those with ratings above the means for feasibility and importance) are prioritized to develop supporting materials in an implementation toolkit. To reduce the potential for our implementation toolkit planning to influence the identification of implementation strategies, we selected implementation strategies for the implementation toolkit based on suggestions from GARDE partners, including their independent importance and feasibility rankings. Data were analyzed and figures were created using R [47] with the following packages: dplyr [48], ggrepel [49], officer [50], flextable [51], and ggplot2 [52]. The R code used to develop the scatterplot was generated with the assistance of Microsoft Copilot. Code was reviewed and validated.

## Results

### Focus Group Discussions/interviews

Seventeen individuals from five healthcare systems participated in a focus group discussion or interview. Focus group discussions/interviews occurred August 12 – September 25, 2024; their duration was 24–58 minutes. No demographic data were collected from participants. Transcripts were analyzed October 14, 2024 – February 18, 2025. Illustrative quotes from focus groups/interviews are available in Supplementary Materials, Table S2. Analysis and data reporting are based on the ELICIT framework. No invited adults dropped out of the study, three invited participants were unable to attend a focus group discussion or interview because of illness or scheduling constraints. Based on focus group discussions/interviews, we determined the ELICIT life cycle phase of each healthcare system (healthcare system 5 = Planning and Implementation/Adaptation; healthcare systems 1 and 2, Implementation/Adaptation, healthcare systems 3 and 4, Post-operations) using our modified definitions of ELICIT level and life cycle phase (Table S1).

### Healthcare System Level

Regardless of implementation phase, all healthcare systems described the GARDE business case assessment. Developing a compelling business case required that GARDE champions consider competing healthcare system priorities. For example, the healthcare system Principal Investigator (PI) at a site in the Planning and Implementation/Adaptation phase (healthcare system 5) stated, “… my … biggest concern is all the powers that be will be like, “We have to do the [non-GARDE precision medicine implementation project] stuff” and … I’ll be like, “Well guess what? We just looked, did something separate and we had this whole catchment group of patients that that would be great for [non-GARDE precision medicine implementation project] …”

Participants at all sites described competition between GARDE and other institutional priorities as a healthcare system-level implementation barrier. Across implementation life cycles, focus group/interview participants identified healthcare system level implementation facilitators: considering multiple factors important to the healthcare system (e.g., security review, patient care goals), and the critical role of champions. Neither Post-operations phase healthcare system (healthcare systems 3 and 4) commented on healthcare system outcomes assessment (Table S2, Supplementary Materials).

### User Level

When focus group/interview participants described gathering user requirements (the user needs that must be fulfilled for GARDE to be successful) [22], they frequently described the critical role of IT staff. GARDE’s acceptability to IT staff was facilitated by its adaptability. When an IT team did not have the skills/knowledge to perform installation steps, installation delays occurred. Across all life cycles, user acceptability of GARDE was facilitated by presentations made to different governance levels. The only participants to comment on initial user satisfaction assessment worked at a site in the Planning and Implementation/Adaptation phases (healthcare system 5). Regardless of the GARDE implementation phase, no participants described their perceptions of long-term user satisfaction.

### Software Level

Gathering technical requirements (the technical needs that must be fulfilled for GARDE to be successful) [22] focused on ensuring GARDE relied on harmoniously combined software. A technical requirements facilitator for the healthcare system in GARDE Planning and Implementation/Adaptation phase (healthcare system 5) was to connect with healthcare systems that had successfully implemented GARDE. Sites in Implementation/Adaptation and Post-operations phases (healthcare systems 1, 2, and 3) did not describe gathering GARDE technical requirements.

Focus group/interview participants identified various software-level implementation barriers and facilitators. Focus group/interview participants from an Implementation/Adaptation site (healthcare system 2) described cross-team collaboration as a GARDE acceptability facilitator (Table 1). At another Implementation/Adaptation site (healthcare system 1), focus group/interview participants described changing healthcare system requirements over the GARDE technical implementation period as a technical acceptability barrier. Despite the goal of a Post-operations site (healthcare system 4) to ensure GARDE interoperability, participants described unforeseen challenges implementing GARDE. Nonetheless, focus group/interview participants from healthcare systems in the Planning and Implementation/Adaptation phases (healthcare systems 2 and 5) indicated that GARDE implementation had been smooth. Focus group/interview participants from a healthcare system in the Implementation/Adaptation phase (healthcare system 2) felt that real-time support from the study team was a critical GARDE implementation facilitator. Focus group/interview participants from a Post-operations site (healthcare system 3) indicated that GARDE’s previous use of a third-party app and procedures specific to their healthcare system were implementation barriers. Focus group/interview participants from a healthcare system in the Post-Operations phase (healthcare system 4) described neither implementation nor technical outcomes assessments. Only focus group/interview participants at a healthcare system in the Planning and Development/Adaptation phase (healthcare system 5) described technical outcomes assessment. Focus group/interview participants from a Post-Operations healthcare system (healthcare system 3) described a project manager’s work as a GARDE implementation facilitator (Table 1).

**Table 1:** Implementation strategies mapped to ELICIT level

		Implementation Strategy	
ELICIT Level	Implementation Barrier	Suggested by Focus Group/interview participant	Suggested by study team
**Healthcare** **System**			
Business case assessment	• GARDE budget did not include non-IT expenses. • GARDE implementation and business case don’t match institutional/leadership priorities.	• Identify resources to fund an IT champion who can drive implementation. • Tailor business case to leadership. • Provides potential sites with multiple business cases.	• Enumerate roles and responsibilities of team members necessary for GARDE implementation.
Acceptability assessment	• Sites need to obtain numerous approvals for implementation. • Getting the right people (including clinical stakeholders) on board is critical.	• Provide data that support financial, security and clinical case for GARDE. • Develop clear guidance for roles and responsibilities of key team members.	
Implementation factor assessment	• Time necessary to identify who should be involved in installation. • Concerns about GARDE increasing primary care workload and causing patient anxiety.	• Provide sample materials to engage stakeholders (i.e., agenda, emails, ppt presentations). • Provide sample messages that are tailored to patient population.	• Include in Gantt chart estimates of time required of key stakeholders.
Outcome assessment	• Uncertain how to continue to promote GARDE use. • Concerns about GARDE placing strain on EHR system.	• Provide materials that facilitate a phased roll out, including a focus on early adopters. • Make GARDE an adjunct to existing EHR tools.	
**User**			
Requirement gathering	• Some teams have to go beyond normal duties for GARDE to be implemented (e.g. additional IT workload was substantial.) • Specific team members were critical because of pre-existing relationships.	• Include step by step crosswalk between implementation strategies. • Allocate sufficient IT resources. • Hire an EPIC consultant.	• Develop clear guidance for roles and responsibilities of key team members to facilitate identifying appropriate champions within institution.
Acceptability assessment	• IT delays because of gaps in the knowledge and skills necessary for installation. • Institutional concerns about communication with patients.	• Provide different forms to support the different implementation strategies (e.g. Azure, AWS). • Make presentations at multiple governance levels, including patient communication committee.	• Provide materials to implementing sites that support presentations, including tailored patient communications.
Satisfaction assessment	• Institutional concern about patient understanding of genetic testing.		• Provide sites with evidence demonstrating that participants in BRIDGE trial adequately understood genetic testing (e.g. resource library of published articles).
**Software**			
Requirements gathering	• Difficult to determine how to combine all elements of GARDE (CDS Hooks, Epic, AWS, chatbot) to best implement it.	• Connect sites that are considering implementing GARDE with sites that have already implemented it.	• Provide risks and benefits of different implementation strategies.
Acceptability assessment	• Unforeseen implementation challenges require collaboration across teams. • Implementation challenges may result from changing healthcare system requirements. • The technical team had to navigate and address security concerns when implementing GARDE at different sites.	• Provide snapshot of data link action criteria build. • Encourage cross team collaboration.	• Provide materials to facilitate site security review.
Implementation assessment	• Site experienced substantial challenges with technical implementation, (i.e. implementing the algorithm and data sharing within the organization) because of use of third-party apps for the outreach component.	• Create a customer support infrastructure (e.g., an internet forum that is moderated by GARDE collaborators; collaborators review forum regularly and respond to inquiries.)	• Create a forum with resources (e.g. videos) that provide scalable support.
Outcomes assessment	• Difficult to understand and diagnose errors that occur in technical implementation.		• Provide more detailed error messages.

GARDE implementation strategies identified by focus group/interview participants and the study team were mapped to ELICIT levels (healthcare system, user and software) and the IT life cycle phase (planning, development, implementation/adaptation, operation). Additionally, we mapped the implementation barrier (s) that were identified by the focus group/interview participants and which are addressed by the strategy to these levels and phases (Table 1).

### Implementation Strategy Survey

Twenty-eight individuals were invited to participate in the Implementation Strategy Survey, April 2–21, 2025. Two individuals had left their positions; no further attempts were made to invite their participation. Nineteen individuals completed the survey. Twelve respondents participated in biweekly GARDE project meetings; 13 participated in a focus group discussion or interview. One respondent did not indicate the importance of an implementation strategy; two declined to indicate the feasibility of at least one implementation strategy. The mean number of years respondents had been in their positions was 8.3 years.

**Fig. 3:**
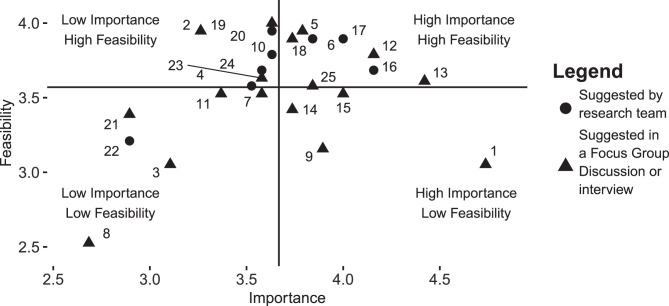
Plot of implementation strategies. Horizontal and vertical lines in the plot indicate the mean values for the importance and feasibility scales. The upper−right quadrant is quadrant 1: relatively high importance and feasibility. The upper−left quadrant is quadrant 2: relatively low importance and relatively high feasibility. The lower−left quadrant is quadrant 3: relatively low importance and feasibility. The lower right quadrant is quadrant 4: relatively high importance and relatively low feasibility. The numbers in the plot map on to the strategies listed in Table 2

Table 2 displays summary statistics for importance and feasibility ratings of each implementation strategy, sorted by the implementation strategy’s quadrant. Figure 3 presents implementation strategies in their quadrants and the group suggesting each strategy.

**Table 2:** Summary statistics for implementation strategy importance and feasibility ratings

Strategy (number represents strategy in Fig. 3)	Importance (Mean ± SD)	Feasibility (Mean ± SD)	Quadrant (Fig. 3)
5. Develop clear guidance for roles and responsibilities of key team members	3.79 ± 1.03	3.95 ± 0.71	1
6. Develop clear guidance for roles and responsibilities of key team members to facilitate identifying appropriate champions within the institution	3.84 ± 0.90	3.89 ± 0.81	1
12. Make presentations at multiple governance levels, including patient communication committee	4.16 ± 0.76	3.79 ± 0.71	1
13. Provide data that support financial, security, and clinical case for GARDE	4.42 ± 0.69	3.61 ± 0.98	1
16. Provide materials to facilitate site security review	4.16 ± 0.83	3.68 ± 0.67	1
17. Provide materials to implementing sites that support presentations, including tailored patient communications	4.00 ± 0.88	3.89 ± 0.81	1
18. Provide sample materials to engage stakeholders (i.e., agenda, emails, ppt presentations)	3.74 ± 0.73	3.89 ± 0.66	1
25. Tailor business case to leadership	3.84 ± 1.01	3.58 ± 0.90	1
2. Connect sites that are considering implementing GARDE with sites that have already implemented it	3.26 ± 0.81	3.95 ± 0.78	2
4. Create a forum with resources (e.g., videos) that provide scalable support	3.53 ± 1.02	3.58 ± 0.90	2
10. Include in Gantt chart estimates of time required of key stakeholders	3.63 ± 1.12	3.79 ± 0.85	2
19. Provide sample messages that are tailored to the patient population	3.63 ± 1.01	4.00 ± 0.82	2
20. Provide sites with evidence demonstrating that participants in the BRIDGE trial adequately understood genetic testing (e.g., resource library of published articles)	3.63 ± 0.96	3.95 ± 0.97	2
23. Provide potential sites with multiple business cases	3.58 ± 0.90	3.63 ± 0.90	2
24. Provide risks and benefits of different implementation strategies	3.58 ± 0.77	3.68 ± 0.89	2
3. Create a customer support infrastructure (e.g., an internet forum that is moderated by GARDE collaborators; collaborators review forum regularly and respond to inquiries)	3.11 ± 1.29	3.05 ± 1.03	3
7. Encourage cross-team collaboration	3.58 ± 1.02	3.53 ± 0.90	3
8. Hire an EPIC consultant	2.68 ± 1.38	2.53 ± 1.22	3
11. Include step-by-step crosswalk between implementation strategies	3.37 ± 0.90	3.53 ± 0.70	3
21. Provide snapshot of data link action criteria build	2.89 ± 0.94	3.39 ± 0.85	3
22. Provide more detailed error messages	2.89 ± 1.10	3.21 ± 0.79	3
1. Allocate sufficient IT resources	4.74 ± 0.45	3.05 ± 1.13	4
9. Identify resources to fund an information technology champion who can drive implementation	3.89 ± 1.05	3.16 ± 1.26	4
14. Provide different forms to support the different implementation strategies (e.g., Azure, AWS)	3.74 ± 0.87	3.42 ± 0.84	4
15. Provide materials that facilitate a phased rollout, including a focus on early adopters	4.00 ± 1.03	3.53 ± 0.70	4

## Discussion

To facilitate the development of an implementation toolkit, this study 1) used an evaluation framework for EHR-integrated innovations (ELICIT) to understand healthcare systems’ experiences implementing GARDE, 2) developed strategies to address barriers to GARDE implementation, and 3) surveyed GARDE partners to evaluate the feasibility and importance of GARDE implementation strategies recommended by focus group/interview participants and GARDE partners. We modified ELICIT for this study (Table S1).

Our findings largely align with previous research on implementing other EHR-integrated innovations. For example, previous research has noted the importance of demonstrating the financial, clinical, and security benefits of health IT systems for gaining institutional support [17, 18]. Our results suggest these benefits may need to be demonstrated to healthcare system leadership. Like Kukhareva et al. (2022), our results suggest that IT and robust technical support are critical to successfully implementing an EHR-integrated innovation [22]. Meeks et al. (2014) found that some dimensions of their sociotechnical model were better represented than others in their data, which is like our results: neither all ELICIT software levels, nor all levels of outcomes/long-term satisfaction assessments were uniformly represented [53]. Nonetheless, relying on ELICIT to interpret our data facilitated tailored implementation strategy development that reflects implementers’ experiences.

The importance and feasibility ratings of implementation strategies in this study were similar to Waltz et al.’s (2015) findings [39]. Implementation strategies related to “Adapt and tailor to context” (e.g., “Tailor business case to leadership”), and “Train and educate stakeholders” (e.g. “Make presentations at multiple governance levels, including patient communication committee”) were rated as both important and feasible. Nonetheless, the ratings revealed nuances that highlighted EHR-integrated innovation funding challenges, including the fact that the strategy with the highest importance rating had a low feasibility rating (“Allocate sufficient IT resources”, Table 2 and Fig. 3), and two Quadrant 4 strategies involved IT funding. Strategies in Quadrant 1 were focused on providing partners with resources (e.g. “clear guidance for roles and responsibilities”, materials and data that supported GARDE) to facilitate implementing GARDE within their institution.

Although there were similarities in our findings and previous research on implementing EHR-integrated innovations, there were also differences. For example, a focus group/interview participant from a healthcare system at the Planning and Implementation/Adaptation phase (healthcare system 5) was concerned that a mismatch between healthcare system priorities may prevent GARDE from moving to a Post-operations phase (Table S2). A scoping review of EHR-integrated technologies for medication-related outcomes found that institutional factors influenced implementation. However, they did not describe institutional *priorities* as an influential factor [54]. The authors have developed multiple business cases that will be included in an implementation toolkit to support implementers in tailoring a business case to address institutional priorities. GARDE has been implemented on both research and clinical bases. We anticipate business cases will vary based on whether implementation is clinical- or research-based.

Organizational readiness and culture, which were identified in a scoping review as influencing implementation of EHR-integrated technologies [54], were not explicitly mentioned in our focus group discussions/interviews. Our group has previously reported on the importance of workflow integration for successfully implementing EHR-integrated technologies [55]. Concerns regarding GARDE integration with existing workflows were not raised by focus group/interview participants This may result from the preponderance of non-clinicians participating in our discussions or the incorporation of workflow considerations in GARDE development [56]. Additionally, although Waltz and colleagues found that nine strategies within the cluster “Develop stakeholder interrelationship” were ranked in Quadrant 1 [39], a strategy that would likely be part of that cluster (i.e., “Facilitate cross team collaboration”), was ranked low importance and feasibility by GARDE implementation partners. Developing partner interrelationships may be less important in implementing EHR-integrated innovations than in implementing other health services.

We found that healthcare systems implementing GARDE had similar experiences (e.g., the importance of making a business case for GARDE), even though the GARDE components that were implemented varied by healthcare system and the infrastructure to support EHR-integrated innovations varied by healthcare system. Regardless of the institutional support for GARDE implementation, healthcare systems were often challenged by IT issues, including relying on individuals not paid through a grant to facilitate implementation. This challenge would be addressed by the implementation strategy of allocating sufficient IT resources (rated as the most important implementation strategy, Table 2). However, this implementation strategy was rated as below the mean feasibility, suggesting that the challenge of identifying and allocating sufficient resources for implementing an EHR-integrated innovation may be a significant barrier to its implementation.

Variability in institutional support and the reliance on volunteer work by IT professionals is reflected in the literature. For example, Sittig and Singh (2010) noted that the success of health IT implementations often depends on the availability of dedicated resources and staff [57]. In our data, implementation strategies that might improve IT support (e.g., “Allocate sufficient IT resources,” “Identify resources to fund an information technology champion who can drive implementation”) were rated as high importance and low feasibility. Their low feasibility rating may result from the need for additional funding [58, 59]. To facilitate sustainable IT support, we are refining an implementation toolkit that will include materials for security review and data that support the financial, security, and clinical case for GARDE. In addition, GARDE deployment may benefit from approaches that simplify IT requirements and shift tasks from EHR IT staff towards research IT resources (such as Clinical and Translational Science Award grantees).

Successful implementation strategies may vary by the life cycle phase of an EHR-integrated innovation, we believe this is an interesting topic for future research. Tailoring an implementation toolkit so that it reflects the life cycle phase of an EHR-integrated innovation may facilitate testing implementation strategies that are specific to the unique needs of a healthcare system.

This study has several limitations. We chose not to map recommended implementation strategies to results from the Expert Recommendations for Implementing Change (ERIC) project [60]. This may limit the comparability of our investigation to other implementation strategy research. However, unlike the ERIC implementation strategies, which were developed to be used across contexts, the implementation strategies we present were specific to an EHR-integrated innovation. The sample for the focus group discussions/interviews was limited to five academic medical centers, which may not represent all healthcare systems that implement GARDE. When analyzing data from focus groups/interviews, we did not analyze by GARDE life cycle, but looked across all healthcare systems because we had few healthcare systems in each life cycle. Although GARDE is an EHR-integrated innovation, our findings may not generalize to all EHR-integrated innovations. It is possible that focus group/interviewee roles and other characteristics (e.g. gender, number of years within an organization) might influence their perspectives on an innovation and its implementation strategies. Unfortunately, we did not ask informants to share that information, so cannot consider its role in this study. Finally, most respondents to the Implementation Strategy Survey were biweekly GARDE meeting attendees involved in GARDE implementation. Although some of these individuals also participated in a focus group discussion/interview, there were fewer respondents from healthcare systems 3 and 5 (ELICIT phases Post-operation and Planning and Implementation/Adaptation). Surveys relied on self-reported data, which may be subject to bias.

## Conclusions

Our focus group discussions/interviews with healthcare systems that have implemented or are implementing GARDE highlight variability in institutional support, the importance of IT support, and making a strong business case for success. The implementation strategies evaluated by GARDE implementers provide valuable insights for future implementations of EHR-integrated innovations. Developing an implementation toolkit based on these findings will support new healthcare systems in implementing GARDE across life cycles and facilitate GARDE’s dissemination.

## Electronic supplementary material

Below is the link to the electronic supplementary material.

Supplementary material 1

## Abbreviations

AWS: Amazon Web Services
BRIDGE: Broadening the Reach, Impact, and Delivery of Genetic Services
CDS: Clinical decision support
EHR: Electronic health record
ELICIT: Evaluation in Life Cycle of Information Technology
ERIC: Expert Recommendations for Implementing Change
IT: Information technology
MUSC: Medical University of South Carolina
NYULH: New York University Langone Health
OSUWMC: The Ohio State University Wexner Medical Center
WCM: Weill Cornell Medicine
UTHealth: University of Utah Health
SD: Standard deviation

## Declarations

### Data availability

The datasets generated and/or analyzed during the current study are not publicly available due to concerns regarding the privacy of individuals who participated in the study. The data will be shared on reasonable request to the corresponding author.
