# Supplementary material for: An exploratory mixed-methods study using the ELICIT framework to identify stakeholder-informed implementation strategies for a population health platform
Source: BMC Health Serv Res. 2026 May 25;26:880. doi: 10.1186/s12913-026-14723-8 (PMC13317325; doi:10.1186/s12913-026-14723-8)
Supplement: Supplementary file 1 — Supplementary material 1 [file 12913_2026_14723_MOESM1_ESM.docx]

INTERVIEW GUIDE

Thank you for participating in our interview today. This interview is being conducted as part of the GARDE research project. The purpose of this interview is to understand more about the GARDE implementation process. Your identifying information will be kept confidential. Do we have your permission to record the meeting today? Great, thank you!

To begin, please say your name and describe your role in GARDE implementation.

1. Please describe where your organization is in terms of GARDE implementation.
2. Please think back to how your organization got to this stage of GARDE implementation. Can you describe the steps you took to implement GARDE, starting with how you first heard about GARDE?
3. How is your organization planning to use GARDE?

| Question | Notes |
| --- | --- |
| *What factors were considered important to assure acceptance of GARDE to clinicians and care coordinators/genetic counselors (i.e., would minimize resistance/disruption and/or maximize its acceptability and feasibility)?*  *What factors were considered important to assure acceptance of GARDE to patients (i.e., would maximize its acceptability and* ***reach****)?* |  |
| *To what extent has GARDE been implemented according to plan?*  *To what extent has the plan needed to be modified?*  *Why? Describe process for modifying the plan (how need for modification was discovered; how did this change impact timeline, design, or other factors)* |  |
| *Could you describe for me the challenges that still exist for using GARDE?* |  |
| What are the costs (personnel and non-personnel) that you encountered getting GARDE up and running?  What are the costs that you encountered during implementation (barriers/facilitators).  What are the anticipated costs for  maintaining GARDE? Who will pay for this?  To what extent do decision makers prioritize the potential downstream revenue associated with programs like GARDE? |  |
| *Do you intend to use GARDE after the completion of this project? Please describe.*  *Do you anticipate any barriers or threats to maintaining GARDE? Please describe.* |  |
| *Throughout the project, we offered technical assistance and designed materials for you to use in GARDE implementation.*  *What did you like about the technical assistance and materials?*  *What did you not like?*  *Since starting to implement GARDE, what additional resources have you sought out to use GARDE?* |  |
| *We are planning to work with other health systems to use GARDE. What recommendations do you have for us about helping other groups to implement and use GARDE?* |  |
| *We’ve talked a lot about GARDE. What other thoughts do you have that we haven’t addressed?* |  |

Table S1. Definition of ELICIT level and lifecycle phase as implemented in this study.

|  |  | ELICIT Lifecycle Phase | | | |
| --- | --- | --- | --- | --- | --- |
| ELICIT level | Defined for this study | Planning – GARDE use is conceived, prioritized and decided upon. | Development– Governance approval is secured; GARDE deployment decisions are made. | Implementation / Adaptation — Implementation is planned, and GARDE is gradually rolled out within HCS. | Operation—GARDE is maintained, updated as needed and disseminated to other HCSs. |
| Society | Health care system (HCS): An organization that includes at least one hospital and at least one group of physicians, all of which use a single EHR, who are connected with each other and with the hospital through common ownership or joint management.^36^ (Adapted) | Process of creating business case for HCS leadership, including discussion of cost | Understanding of organizational acceptability of implementing GARDE. | Description of the process of assessing HCS factors necessary for successful implementation. | Discussion of long-term outcomes for the HCS related to GARDE implementation. |
| User | Physician, genetic counselor, genetic counseling assistant, patient, Information Technology (IT) / Information Security (IS) personnel | Mention of specific requirements for users | Assessment of usability including ease of use, understanding, and learning | Assessment of satisfaction when system is being used | User long term satisfaction |
| Software | GARDE, including the Population Health Management dashboard and chatbot | Discussion about the process of gathering technical resources/needs– quality of software | Discussion about GARDE technical and security requirements; how they were identified and addressed. Fit between GARDE technical aspects and HCS. | Quality of information provided during implementation | GARDE’s interoperability and integration throughout HCS. |

Table S2: Illustrative quotes by healthcare system according to each ELICIT Phase and Level.

|  | **ELICIT Life Cycle Phase** | | | | |  |
| --- | --- | --- | --- | --- | --- | --- |
|  | **Planning and Implementation / Adaptation** | **Implementation / Adaptation** | **Implementation / Adaptation** | **Post-operations** | **Post-operations** | |
| **ELICIT Level** | **HCS** **5** | **HCS** **1** | **HCS** **2** | **HCS** **3** | **HCS** **4** | |
| **HCS** |  |  |  |  |  | |
| Business case assessment | “Fortunately, GARDE is being done outside electronic health record, but all the other EHR implementation projects genetics have been involved with, everything was put on hold. We have to do [other precision medicine implementation project].  And so probably…my biggest concern is all the powers that be will be like, “We have to do [non-GARDE precision medicine implementation project] stuff” and…I'll be like…”Guess what. We just looked, did something separate, and we had this whole catchment group of patients that that would be great for [non-GARDE precision medicine implementation project]” and it's just that nobody really pays attention.” HCS PI | “I didn't really budget anything for more of the clinical side….I was really heavily thinking about the technical integration and implementation and not so much…getting people into the care.” HCS PI | “…*paying* for time essentially for…local IT champion would make things…move a little more swiftly and smoothly.” HCS PI | “…I've experienced both sides of the spectrum. I've been told, “It's not tangible. It's not real.” Whatever. “We can't, we can't put our finger on that.” And then I, I've seen and experienced like, “This is all the downstream revenue we must focus on that.” So it does seem to be very individualized, depending on who up within the system is looking at that.” Lead genetic counselor | “Did they want us seeing patients in their clinic as a way to bring people physically into that building and potentially support other services?...We’ve had those [cost] conversations internally as a team…” Lead genetic counselor | |
| Acceptability assessment | “And then internally it took us probably three or four months to get the right players together to convince them that this would be an important next step and then it … finally was given approval to move ahead” HCS PI | “…a lot of [HCS] patients do not have a PCP listed. And one of the things I've been talking with our primary care leadership about is actually making a referral …from people that are going through GARDE, back into primary care.” HCS PI | “…*clearly* this is a financial no brainer and that's frankly what motivates healthcare systems more than anything else and so… doing cancer risk stratification, getting more procedures, getting more… evidence-based patient-centered ways....So this met every single checkbox.” HCS PI | “…even in the wording for our messages, it was like “we're collaborating with your primary care doctor and *they recommend*,”…it was… to…show that we aren't just random people looking at your records and things like that.” Lead genetic counselor | “Navigating governance was always top of our minds and it’s something I remember as we started working with <<HCS 6>> and <<HCS 3>>.” GARDE PI | |
| Implementation assessment |  |  | “…there were subsequent delays in establishing what team would be extracting the necessary data to run GARDE.  Would this be coming from the <<>> team? So you know, the people granting us approval to… run GARDE…on our data or would the <<HCS 02>> team locally extract data?” HCS PI | “…[W]e give a lot of thought about this before rolling out…how should we engage providers?" Project manager | “Biggest concern, certainly to primary care, was anxiety. They were *very* worried that those would cause a lot of anxiety with their patients…we didn’t see any evidence for that…in the electronic health record…[or] anecdotally from patients” GARDE PI | |
| Outcomes assessment | “It would be kind of cool to do a multi center collaborative effort with all of this, right? So some type of project, you know, especially if all centers were…up in all two phases [PHM and chatbot]…and we were able to do some type of comparative project or some type of larger scope project.” HCS PI | “…I would think from an IT side…they would look at that, what's the system usage? How's it…impacting the system? As far as other metrics…I don't know what they would look at that would just be …from my experience what…I felt like people were looking at...” Senior Epic Analyst | “…I don't imagine it would be installed institution-wide immediately, but I…expect this…will…serve as an adjunct to existing tools….... the ultimate aim is for everyone to use it.” Research specialist |  |  | |
| **User** |  |  |  |  |  | |
| Requirement gathering | “…somebody spun up the server and then it came down to OK. Well, who's gonna do what?...So, I guess I kind of volunteered our team or my team to pull the data because we have that expertise and we can automate it and we can load it and all that stuff. That's what our team does. We have a bunch of data engineers so it couldn't have been not just me. It could have been somebody else on our team…that does it. And the next phase, we’re not fully implemented yet, is, you know you get the results and then that those results need to go into Epic.” Information systems analyst | “…we have primary care leadership on board with regards to making sure that they're comfortable with us reaching out to their patients etc. But it wasn't…primary care that initiated the need for GARDE.” HCS PI | “The <<platform> team's job at <<HCS 02>> essentially stops at giving us access to the <<platform>> and facilitate the computer resources, which include creating the allocated space, the disk space, give us CPU access within <<platform>>, create the depository service, the SQL service.” HCS PI | “But on the… informatics side, so <<03-05>> has…particular expertise as a director with our IT department, especially working with Epic and <<they>> obviously <<have>> a close relationship with <<04-05>> and <<04-01>>….So <<they>> and <<their>> team were…our first point of contact for discussing how to get this…GARDE algorithm implemented into Epic and figuring out… what's needed from our end, what other resources we needed.…this process probably took a lot longer than we anticipated….I don't think we knew…going into it how laborious that task would be.” Project manager | “…on the back end…we had several meetings [with IT department personnel] trying to work through the process… through requirements to build that register…took a fair amount of discussion until we got to that space….They worked with <<04-04>> and team…to help figure out the requirements of data elements should be on that registry” GARDE PI | |
| Acceptability assessment | “…spam or something because I don't know anything about it. And I probably won't respond to it at all. So, a lot of that is, you know, that's where I was coming from, 'cause…whenever you communicate with somebody, externally, you have to make sure that you have their consent to send them stuff.” Bioinformaticist | “…there's also the human side of making sure that…clinical staff are on board and ready for implementing or receiving patients that are positive.” HCS PI | “My understanding is that the five…PCPs…that initially agreed to having GARDE run…in this population were quite excited about it and had no qualms whatsoever….this remains to be seen as to how other people…would respond  to this.” HCS PI | “…there's a…few components…even just…the outreach component itself, the way that we worded that initial…outreach engagement message was important. We spent quite a few…days working on that…making sure it's not too much information, that the wording is correct….that in itself is…a really crucial…step, especially with genetics…sometimes being…a polarizing topic, depending on the person.” Project manager | “We did have to go through…various levels of governance…We made presentations to a population health group…the Patient Communication Committee…we had meetings with primary care…I think that was really, *really* critical.” Project PI | |
| Initial user satisfaction assessment | “…a percentage of folks. You know, again, there's concerns about, “is my insurance going to pay for this?...What does this all mean?” You know, we're doing a fairly large panel test and I was sort of coming into breast cancer risk concerns and we're finding that there are increased risk for other types of cancer and sometimes you know that lack of patient understanding.” HCS PI |  |  |  |  | |
| Long-term user satisfaction assessment |  |  |  |  |  | |
| **Software** |  |  |  |  |  | |
| Technical requirements gathering | “Your…group provided contacts and said, “Hey, these are the people we worked with at <<HCS 03>>, or…<<HCS 01>>.” And, and I think that was extremely helpful because… Our folks then reached out to them to and in a way that was even more reassurance and you know what the <<HCS 04>> group knows what they're doing. They're not here to just…take things over.” HCS PI | “We intend to use all the pieces of it…” HCS PI |  |  | “…details like using standard terminologies and things like that and ways to map onto the terminology and evaluating Epic with an eye of…being able to use it for other systems as well….trying to figure out…what in Epic was repeatable versus not repeatable….” Lead software engineer | |
| Acceptability assessment | …so we had three or four conversations,“<<05-02>>, I can see the utility and we can definitely we have the resources to do Phase 1. But we're not gonna do Phase 2 yet. We're first gonna do Phase 1, and then we're gonna sit back and kinda see.” So as far as…incorporating it using MyChart messaging and so on. So as far as clinician involvement, I gotta say <<they were>> concerned that if we had a system in place, an electronic health record, that automatically messaged patients without the provider input. That was gonna be a big issue.” HCS PI | “We had to create a way to get the SDEs that we needed into Epic. And we then created them, but we had to figure it out. Well, we figured it out once…in the meantime, the policy has changed and we no longer have to use that gateway. So now we're having to figure out how to get them in a different way. And this is…the challenge of…a project taking place over the course of months.” Bioinformatician | “Beyond that I do suspect there would be technical challenges…to installing the chatbot….And of course, there's certainly the small possibility that this, that this goes through without challenges.” HCS PI | “…once we got the project manager, we were then able to sort of go into…the bowels of our institution’s…Epic, to figure out…”Can this be easily integrated?” And the answer is no….as much as…<HCS 04>> wanted it to be…a one size fits all plug and play it just…wasn't the case and I think from that it unearthed a lot of understanding that even though Epic is a huge…. it just isn't the same across any institution.” Project manager | “The other part of it too, is the interoperability strategy . . . understanding the security concerns of others and what they were hoping they would do versus what they really would do were not exactly the same.” Lead software engineer | |
| Implementation assessment | And yeah, my understanding is overall… and really thanks to your team's responsiveness too. I…should say <<04-02>> and the whole group has been extremely responsive. So I'm…*really* happy because I've been involved with other IT projects…and sometimes…I have to bug people like “well, where are things at?”...there's a constant, really good communication back and forth between the two groups, the two centers…. So my understanding is that things are moving along very smoothly in the whole scheme of things and still on a timeline. HCS PI | “Let's start with the install….I don't think that it was completely solidified. There were different versions of GARDE coming out during the time…when we were installing it…and we installed it on desktops and on the server, and, and all that. So we had…lots of install experience…and they were all a little bit different. They all required a few different commands. Hopefully that has solidified now and we have more of a…process…” Bioinformatician | “…I don't think GARDE could have been successfully installed…without the support. The biggest thing I think was just direct support from <<04-02>> and <<04-01>> through multiple meetings and, and Zoom calls…. personally, in my experience, and I'm pretty sure I can speak…to <<02-03>>'s experience as well…we didn't use written materials nearly as much as we did…direct access and several Zoom calls and meetings to troubleshoot and problematize along the way.” HCS PI | “…in terms of being able to integrate a third-party app or API with Epic, that part was difficult because that required our side to do this whole, huge effort to give every single person…a psuedo MRN…just the limited amount of information that we could give to <<3^rd^ party chatbot developer>> and…what our institution allowed and just being able to make everything secure and…housing it in…<<cloud platform>> and…what does that information being translated look like and…the back and forth communication with…technology that part took a little bit to like hammer out...” Project manager |  | |
| Outcomes assessment | “…diagnosing issues instead of like digging. Obviously, yes, you can dig into the logs, but it'd better if there's more information. If you want people to use the UI [user interface] that you can look at it there, or if there's a way to actually look at the log files…it's just been…a struggle…there's an error with loading this data. “OK, I don't know what it is 'cause the error message wasn't very clear. OK, well, I gotta piece this out.” Bioinformatician |  |  |  |  | |
